# Risk factors for re-expansion pulmonary edema following chest tube drainage in patients with spontaneous pneumothorax: A systematic review and meta-analysis

**DOI:** 10.34172/jcvtr.32871

**Published:** 2024-03-13

**Authors:** Herick Alvenus Willim, Eva Lydia Munthe, Yoni Vanto, Alvin Ariyanto Sani

**Affiliations:** ^1^Dr. Agoesdjam Regional Public Hospital, Ketapang Regency, West Kalimantan, Indonesia; ^2^Department of Pulmonology and Respiratory Medicine, Dr. Agoesdjam Regional Public Hospital, Ketapang Regency, West Kalimantan, Indonesia; ^3^Department of Surgery, Dr. Agoesdjam Regional Public Hospital, Ketapang Regency, West Kalimantan, Indonesia; ^4^Department of Cardiothoracic and Vascular Surgery, Dr. Soedarso Regional Public Hospital, Pontianak, West Kalimantan, Indonesia

**Keywords:** Re-expansion pulmonary edema, Chest tube, Spontaneous pneumothorax

## Abstract

Re-expansion pulmonary edema (RPE) is a rare but potentially life-threatening complication that can occur after rapid lung expansion following the management of lung collapse. This meta-analysis aimed to investigate the risk factors for RPE following chest tube drainage in patients with spontaneous pneumothorax. We conducted a comprehensive systematic literature search in electronic databases of PubMed, ScienceDirect, Cochrane Library, and ProQuest to identify studies that explore the risk factors for RPE following chest tube drainage in spontaneous pneumothorax. Pooled odds ratios (OR) or weighted mean differences (WMD) were calculated to evaluate the risk factors. Statistical analysis was conducted using Review Manager 5.3 software. Five studies involving 1.093 spontaneous pneumothorax patients were included in this meta-analysis. The pooled analysis showed that the following risk factors were significantly associated with increased risk of RPE following chest tube drainage: the presence smoking history (OR=1.94, 95% CI: 1.22-3.10, *P*=0.005, I2=0%), longer duration of symptoms (WMD=3.76, 95% CI: 2.07-5.45, *P*<0.0001, I2=30%), and larger size of pneumothorax (WMD=16.76, 95% CI: 8.88-24.64, *P*<0.0001, I2=78%). Age, sex, and location of pneumothorax had no significant association. In patients with spontaneous pneumothorax, the presence of smoking history, longer duration of symptoms, and larger size of pneumothorax increase the risk of development of RPE following chest tube drainage.

## Introduction

 Spontaneous pneumothorax is a condition characterized by the sudden presence of air within the pleural space without apparent external cause, which may result in lung collapse and impaired respiratory function.^1^ It is one of the common cases encountered in the emergency department. Chest tube drainage is a common intervention for the management of pneumothorax, which involves the insertion of a tube into the pleural space to evacuate the air and allow the lung to re-expand. Although chest tube drainage is generally considered a safe and effective procedure, a small number of cases may develop a rare but potentially fatal complication known as re-expansion pulmonary edema (RPE).^2^

 RPE can develop after rapid reinflation of a collapsed lung due to pleural effusion, atelectasis, or pneumothorax.^3^ The underlying mechanism of RPE is not fully understood, but it is thought to be due to a combination of increased capillary permeability, inflammation, and altered lymphatic drainage.^4^ The clinical presentation of RPE can range from mild respiratory distress to severe hypoxia and respiratory failure. Typically, the symptoms appear within the first hour after lung re-expansion following thoracocentesis or chest tube drainage and worsen in intensity over the next 24-48 hours.^5^

 The incidence of RPE is reported to occur in less than 1% of spontaneous pneumothorax following chest tube drainage. Despite its rarity, RPE is a serious complication that can lead to significant morbidity and mortality.^6^ As such, it is important to identify the risk factors that may increase the likelihood of developing RPE following chest tube drainage in this population. To address this issue, we conducted a systematic review and meta-analysis of the available literature to identify the risk factors for RPE following chest tube drainage in patients with spontaneous pneumothorax. Knowing the risk factors may help clinicians identify high-risk patients and implement preventative measures to reduce the incidence of this life-threatening complication.

## Materials and Methods

###  Study Registration

 This study protocol was registered in the International Prospective Register of Systematic Reviews (PROSPERO) with the registration number of CRD42023404771.

###  Search strategy

 This meta-analysis was conducted according to the Preferred Reporting Items for Systematic Reviews and Meta-Analyses (PRISMA) guidelines.^7^ We conducted a comprehensive systematic literature search of computerised databases including PubMed, ScienceDirect, Cochrane Library, and ProQuest to identify eligible studies published up to February 28, 2023. The search terms used were combination of the following keywords: (“risk factors”) AND (“re-expansion pulmonary edema” OR “RPE”) AND (“chest tube”) AND (“spontaneous pneumothorax” OR “pneumothorax”). There was no country restriction. In addition, we also manually searched the references of the relevant papers for potential additional articles.

###  Inclusion and exclusion criteria

 The inclusion criteria for eligible studies were as follows: 1) cross-sectional, case-control, retrospective, or cohort studies; 2) patients undergoing chest tube drainage for spontaneous pneumothorax; 3) evaluated risk factors of re-expansion pulmonary edema; 4) sufficient data to obtain the number of events needed for computing odds ratios (OR) or weighted mean differences (WMD) and the corresponding 95% confidence intervals (CI); 5) available as full-text studies. The exclusion criteria were as follows: 1) case reports, review articles, letters, editorials, and conference abstracts; 2) animal studies; and 3) studies with insufficient data for the estimation of effect size; and 4) duplicate or overlapping studies.

###  Data extraction

 The data extraction of the relevant studies was performed independently by two authors (HAW and ELM). Any disagreement about data extraction was resolved by discussion with the third (YV) and fourth (AAS) author. We collected several details from the selected articles, such as the name of first author, publication date, study location, study design, demographic characteristics, sample size (in either RPE or non-RPE group), risk factors, and necessary data for calculating OR or WMD and 95% CI.

###  Quality assessment

 The quality of the included studies were independently assessed by two authors (HAW and EVL). Any disagreement was resolved by discussion with the third (YV) and fourth (AAS) author once again. The Newcastle-Ottawa Scale (NOS) was used to assess the quality of the included studies.^8^ The NOS has a total score of 0 to 9 stars based on three criterias: patient selection (0 to 4 stars), comparability of study groups (0 to 2 stars), and outcome (0 to 3 stars). Studies with a total score of ≥ 7 were regarded as high quality studies, 5 to 6 were regarded as moderate quality studies, and ≤ 4 were regarded as low quality studies.

###  Statistical analysis

 Meta-analysis was conducted using either WMD for continuous variables or OR for dichotomous variables. The heterogeneity was assesed using the Cochran’s Q Chi-square test and I^2^ statistic. If the P value was below 0.05 and I^2^ was greater than 50%, the heterogeneity is considered significant. In cases where there was no heterogeneity, we utilized a fixed-effects model approach. However, if there was heterogeneity, we used a random-effects model instead. We considered any test statistics with a *P* value less than 0.05 to be statistically significant. The visual funnel plot was used to assess the potential publication bias. Statistical analysis was conducted using Review Manager 5.3 software (The Nordic Cochrane Centre, Copenhagen).

## Results

###  Literature search

**Figure 1:**
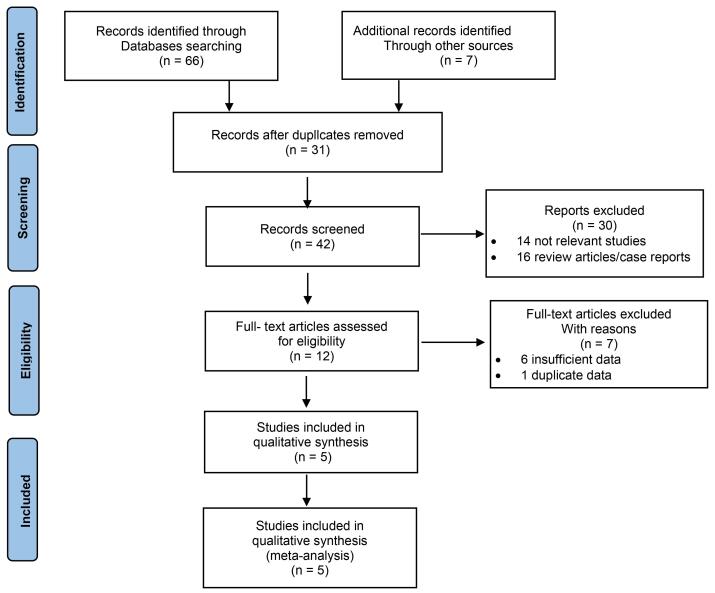
Literature search flow chart

 The systematic literature search was conducted on electronic databases, resulting in an initial total of 66 potential articles. Additionally, 7 articles were found by manually searching relevant literature. After removing duplicate articles, 42 articles were screened based on the titles and abstracts. From these, 12 articles were selected for full-text review and 7 articles were excluded. Ultimately, 5 studies were included in the meta-analysis. A flowchart of the literature search process is presented in Figure 1.

###  Study characteristics and quality assessment

 This meta-analysis comprised of five studies involving 1.093 patients.^9,10,11,12,13^ All studies were retrospective cohort studies, conducted between 2011 and 2014. Two studies were conducted in Korea^9,12^ and three in Japan.^10,11,13^ The mean age of participants ranging from 27 to 48 years old. The NOS was employed to assess the quality of the studies, with the quality scores ranging from seven to eight, indicating a generally high study quality. Table 1 presents a summary of the included studies.

**Table 1:** Characteristics of the included studies in the meta-analysis

**First author, year**	**Country**	**Design**	**Sample size**	**Mean Age (years)**	**Male, n (%)**	**Patients with RPE, n (%)**	**NOS score**
Kim, 2011^9^	Korea	RC	112	32	87 (77.7)	22 (19.6)	7
Haga, 2013^10^	Japan	RC	462	40	369 (79.9)	30 (6.5)	8
Morioka, 2013^11^	Japan	RC	173	48	148 (85.5)	27 (15.6)	7
Yoon, 2013^12^	Korea	RC	306	44	262 (85.6)	49 (16.0)	8
Taira, 2014^13^	Japan	RC	40	27	32 (80.0)	13 (32.5)	7

Abbreviations: NOS, Newcastle-Ottawa Scale; RC, retrospective cohort; RPE, re-expansion pulmonary edema. *P*< 0.05 statistically significant.

###  Pooled results

 The pooled results of included studies are shown in Table 2.

**Table 2:** Meta-analysis of risk factors for re-expansion pulmonary edema following chest tube drainage in spontaneous pneumothorax

**Risk factor**	**Studies**	**I**^2^** (%)**	**OR or WMD**	**95% CI**	***P*** ** value**
Age	5	0	2.83	-0.43 - 6.10	0.09
Sex	3	18	0.86	0.46 - 1.61	0.63
Smoking history	3	0	1.94	1.22 - 3.10	0.005
Location of pneumothorax	2	0	1.38	0.78 - 2.43	0.27
Duration of symptoms	4	30	3.76	2.07 - 5.45	< 0.0001
Size of pneumothorax	4	78	16.76	8.88 - 24.64	< 0.0001

*P* < 0.05 statistically significant.

###  Age

**Figure 2:**
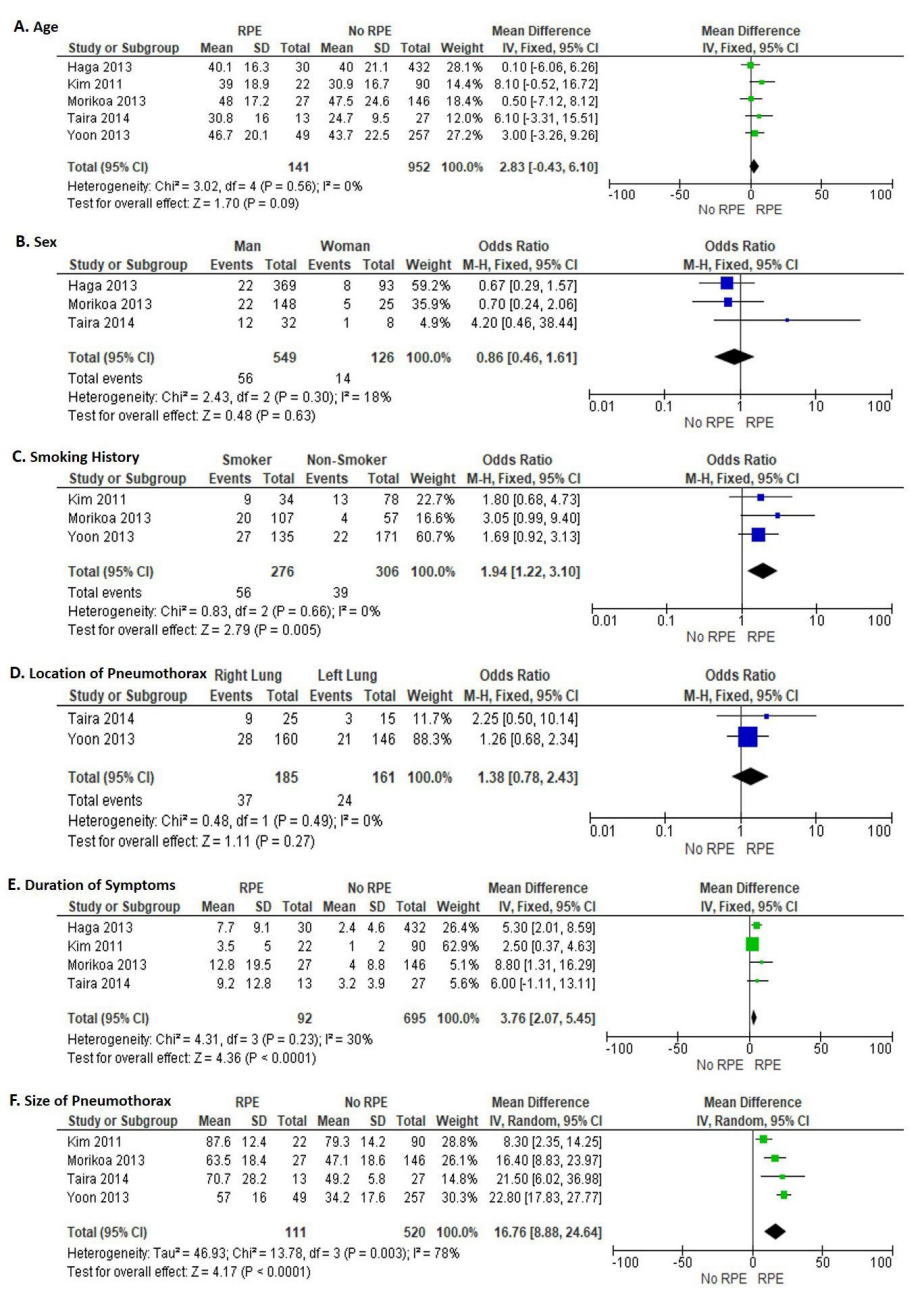
Forest plots showing the results of the meta-analysis of risk factors for re-expansion pulmonary edema following chest tube drainage in patients with spontaneous pneumothorax: (A) age, (B) sex, (C) smoking history, (D) location of pneumothorax, (E) duration of symptoms, (F) size of pneumothorax

 Five studies reported data for age. No significant heterogeneity was found in the studies (I^2^ = 0%; *P* = 0.56). The pooled analysis using a fixed-effects model revealed that age was not significantly associated with RPE following chest tube drainage in patients with spontaneous pneumothorax (WMD = 2.83; 95% CI = -0.43 - 6.10; *P* = 0.09; Figure 2A).

###  Sex

 Three studies reported data for sex. No significant heterogeneity was found in the studies (I^2^ = 18%; *P* = 0.30). The pooled analysis using a fixed-effects model revealed that sex was not significantly associated with RPE following chest tube drainage in patients with spontaneous pneumothorax (OR = 0.86; 95% CI = 0.46 - 1.61; *P* = 0.63; Figure 2B).

###  Smoking history

 Three studies reported data on smoking history. No significant heterogeneity was found in the studies (I^2^ = 0%; *P* = 0.66). The pooled analysis using a fixed-effects model revealed that smoking history was significantly associated with RPE following chest tube drainage in patients with spontaneous pneumothorax (OR = 1.94; 95% CI = 1.22 - 3.10; *P* = 0.005; Figure 2C). This result indicates that smoking increases the risk of RPE.

###  Location of pneumothorax

 Two studies reported data for the location of pneumothorax. No significant heterogeneity was found in the studies (I^2^ = 0%; *P* = 0.49). The pooled analysis using a fixed-effects model revealed that location of pneumothorax was not significantly associated with RPE following chest tube drainage in patients with spontaneous pneumothorax (OR = 1.38; 95% CI = 0.78 - 2.43; *P* = 0.27; Figure 2D).

###  Duration of symptoms

 Four studies reported data for the duration of symptoms. No significant heterogeneity was found in the studies (I^2^ = 30%; *P* = 0.23). The pooled analysis using a fixed-effects model revealed that duration of symptoms was significantly associated with RPE following chest tube drainage in patients with spontaneous pneumothorax (WMD = 3.76; 95% CI = 2.07 - 5.45; *P* < 0.0001; Figure 2E). This finding indicates that patients with longer symptoms of pneumothorax have an increased risk of RPE.

###  Size of pneumothorax

 Four studies reported data for the size of pneumothorax. There was significant heterogeneity found in the studies (I^2^ = 78%; *P* = 0.003). The pooled analysis using a random-effects model revealed that size of pneumothorax was significantly associated with RPE following chest tube drainage in patients with spontaneous pneumothorax (WMD = 16.76; 95% CI = 8.88 - 24.64; *P* < 0.0001; Figure 2F). This finding indicates that patients with larger size of pneumothorax have an increased risk of RPE.

###  Publication bias

 The funnel plot cannot be performed due to the limited number of studies available for each risk factor, which requires a minimum of ten studies.

## Discussion

 This systematic review and meta-analysis included five retrospective cohort studies involving a total of 1,093 patients with spontaneous pneumothorax treated by chest tube drainage. Our results revealed that in patients with spontaneous pneumothorax, the presence of smoking history, longer duration of symptoms, and larger size of pneumothorax increase the risk of development of RPE following chest tube drainage. To the best of our knowledge, this is the first meta-analysis that examines the risk factors for RPE following chest tube drainage in patients with spontaneous pneumothorax.

 The exact pathophysiology underlying RPE remains unclear. It involves a complex interplay of various factors. One proposed mechanism is the reperfusion injury that occurs when a collapsed lung is rapidly re-inflated. This reperfusion can lead to increased pulmonary capillary permeability, which can result in an inflammation response and the production of oxygen-derived free radicals.^14^ Another potential mechanism is the increased hydrostatic pressure that results from the greater venous return to the lungs during re-expansion. This pressure can lead to mechanical damage to alveolar capillaries and further increase pulmonary capillary permeability. Furthermore, lymphatic clearance of fluid from the lungs may also be impaired during lung collapse, leading to an accumulation of fluid that can contribute to the development of RPE.^15^

 Our meta-analysis showed that smokers have nearly twice the risk of developing RPE compared to non-smokers. Smoking is known to cause chronic inflammation and lung injury, which can worsen the underlying mechanisms that lead to RPE. In smokers, the lungs may have decreased compliance and increased airway resistance due to inflammation and narrowing of the airways, making it less able to cope with the sudden changes in pressure and blood flow that occur during re-expansion after chest tube drainage.^16^ Moreover, smoking can lead to increased capillary permeability and impaired gas exchange in the lungs, making it harder for the lungs to remove excess fluid and maintain appropriate gas exchange in response to sudden changes in pleural pressure. These factors make smokers more susceptible to RPE following chest tube drainage.^17^

 The duration of symptoms indicates how long a collapsed lung has occurred. More than 80% of cases of RPE occur in patients with prolonged lung collapse (3-7 days). An animal experimental study by Sewell et al demonstrated changes in the alveolar-capillary basement membrane occuring 72 hours after lung collapse, resulting in increased membrane permeability.^18^ Another experimental study by Miller et al demonstrated that RPE only occurred in the group with a collapsed lung for more than 3 days.^19^ Our meta-analysis results are consistent with these findings, as the RPE group had a mean difference in the duration of symptoms that was more than 3 days longer than the non-RPE group. During collapse, several changes can occur in the lung, including a decrease in surfactant and regional tissue hypoxemia. During rapid re-expansion of the lung, blood flow increases and the alveoli rapidly expand, causing mechanical alveolar injury. This injury triggers an inflammatory response and an increase in free radicals, which can lead to increased permeability of the pulmonary vessels. Consequently, fluid and protein can leak into the alveoli, resulting in pulmonary edema. ^20,21^

 Size of pneumothorax is a strong predictor of RPE. When the lung collapses due to a pneumothorax, the volume of air in the pleural space has been linked to the risk of RPE. Larger pneumothorax is more likely to be complicated by RPE. A study by Matsuura et al classified pneumothorax size as small (1/3 hemithorax), moderate ( > 1/3 hemithorax), and severe (complete lung collapse). The occurrence of RPE in these groups was 0%, 7%, and 17%, respectively, and was observed in nearly half of the cases diagnosed as tension pneumothorax.^22^ In cases of large pneumothorax, the pulmonary microvascular endothelium tends to undergo more thickening and hardening. When re-expansion occurs, the sudden increase in tensile stress leads to more injury in the pulmonary microvessels. This can cause structural and biochemical changes, which may result in inflammation and increased vascular permeability, leading to pulmonary edema.^23^

 Although rare, RPE is important to recognize because it can cause significant respiratory distress and can even be lethal in severe cases with mortality rate up to 20%.^24^ Patients with RPE typically experience a sudden onset of shortness of breath and tachypnea, along with other manifestations such as chest discomfort, cough, and hypoxemia. In severe cases, it can lead to shock and even death. The symptoms of RPE are typically observed within minutes to hours following chest tube drainage, although it may be delayed for up to 48 hours. A chest X-ray of a patient with RPE may show an alveolar filling pattern. The typical finding on a chest computed tomography (CT) scan is the presence of ground-glass opacities in the lungs. Diagnosis of RPE is established based on a high suspicion of clinical deterioration following re-expansion of a collapsed lung, supported by chest imaging.^25,26^

 The management approach of RPE depends on the severity of the condition, with supportive care being the general approach. Oxygen therapy with nasal cannula or face mask may be sufficient for patients with mild symptoms. Noninvasive ventilation with bilevel positive airway pressure may be useful in patients with worsening symptoms. Patients with severe symptoms may require endotracheal intubation and mechanical ventilation. Patients with unilateral pulmonary edema may benefit from lying on the unaffected side to improve intrapulmonary shunt.^27,28^ There are no specific medication guidelines for RPE. Some evidence suggests that the use of diuretics, steroids, and inotropes may be considered based on clinical indications.^29,30,31^ The use of ibuprofen or prostaglandin analogs such as misoprostol for the cytoprotective or anti-inflammatory effects has been reported, but the benefit is still unclear.^32^ Suction should not be routinely given to patients who have these risk factors due to the increased risk of RPE. If needed, the recommended pressure given should be low, between -10 to -20 cm H_2_O.^33^ Most of RPE resolves within a period of 24 to 72 hours.^34^

 The findings of this study have important clinical implications for the management of patients with spontaneous pneumothorax undergoing chest tube drainage. Specifically, our results suggest that clinicians should be aware of the increased risk of RPE in patients with a smoking history, longer duration of symptoms, or larger size of pneumothorax. These patients require more careful monitoring and management during and after chest tube drainage. Additionally, our study highlights the need for further research to better understand the underlying mechanisms of RPE and identify effective strategies for prevention and management of RPE.

 This meta-analysis had certain limitations. First, the studies included in our meta-analysis were only found from Japan and Korea, which may not represent the global population. Second, the number of included studies was relatively small due to limited data availability. Third, publication bias could not be excluded as we could not perform a funnel plot analysis due to the limited number of studies. The number of original studies investigating the risk factors associated with RPE in spontaneous pneumothorax is still limited.

## Conclusion

 This systematic review and meta-analysis revealed that in patients with spontaneous pneumothorax, the presence of smoking history, longer duration of symptoms, and larger size of pneumothorax increase the risk of development of RPE following chest tube drainage. Clinicians should be aware of patients with these risk factors. These high risk patients require more careful monitoring and management during and after chest tube drainage.

## Competing Interests

 The authors declare that there are no conflicts of interest.

## Ethical Approval

 Not applicable.

## Funding

 None.
